# Understanding the pubertal, psychosocial, and cognitive developmental trajectories of stunted and non-stunted adolescents: protocol of a multi-site Indonesian cohort study

**DOI:** 10.3389/fped.2024.1296128

**Published:** 2024-04-16

**Authors:** Bernie Endyarni Medise, Madarina Julia, Yoga Devaera, Mei Neni Sitaresmi, Asmarinah, Nur Aisiyah Widjaja, Royke Tony Kalalo, Frida Soesanti, Dewi Friska, Wani Riselia Sirait, Peter Azzopardi, Susan Sawyer

**Affiliations:** ^1^ Department of Child Health, Faculty of Medicine, Universitas Indonesia, Jakarta, Indonesia; ^2^ Department of Child Health, Faculty of Medicine, Public Health and Nursing, Gadjah Mada University, Yogyakarta, Indonesia; ^3^ Department of Medical Biology, Faculty of Medicine, Universitas Indonesia, Jakarta, Indonesia; ^4^ Department of Child Health, Faculty of Medicine, Airlangga University, Surabaya, East Java, Indonesia; ^5^ Department of Psychiatry, Faculty of Medicine, Airlangga University, Surabaya, East Java, Indonesia; ^6^ Department of Community Medicine, Faculty of Medicine, Universitas Indonesia, Jakarta, Indonesia; ^7^ Faculty of Medicine, Universitas Indonesia, Jakarta, Indonesia; ^8^ Centre for Adolescent Health, Royal Children's Hospital, Melbourne, VIC, Australia; ^9^ Murdoch's Children Research Institute, Melbourne, VIC, Australia; ^10^ Department of Paediatrics, The University of Melbourne, Melbourne, VIC, Australia

**Keywords:** adolescent, behavior, development, growth, mental health, nutrition, puberty, stunting

## Abstract

**Background:**

The prevalence of stunting among Indonesian children aged 5–12 years decreased from 30.7% in 2013 to 23.6% in 2018 but has remained among the highest rates worldwide. Furthermore, Indonesian children were shorter than the standard reported by the World Health Organization and experienced obesity. The Indonesian government has created many programs to reduce stunting in children under the age of 5 years. An early preventive strategy is necessary because stunting can manifest within the initial 1,000 days of life, including during pregnancy. Therefore, a newer perspective, such as that achieved by addressing stunting in adolescents, has been deemed useful, given that adolescents are in their pubertal stage and are undergoing lifestyle changes. This cohort study was designed to measure these factors comprehensively in stunted and non-stunted children as they pass through adolescence.

**Methods:**

For the prospective cohort, 560 individuals will be recruited from DKI Jakarta, DI Yogyakarta, and East Java. The participants will be categorized into stunted and non-stunted groups, then undergo annual examinations in which key objectives, such as weight, height, and body mass index ,will be assessed for the growth profile; waist circumference, middle-upper arm circumference, hand-grip strength, body fat percentage, and food intake will be evaluated in a nutritional assessment; psychosocial and mental issues will be evaluated according to behavioral problems, symptoms of depression, quality of life, sleep patterns, anxiety disorders, and parenting style through the use of specific questionnaires; and pubertal stage will be assessed using a self-report questionnaire. Some cross-sectional data, such as cognitive performance, hair zinc levels, vitamin D levels, bone mineral density, and bone age, will also be included. All the outcomes will be analyzed in accordance with the variable types.

**Discussion:**

This study provides a thorough dataset of Indonesian adolescents encompassing several elements, such as growth, nutrition, psychosocial wellbeing, mental health, and pubertal development, for both stunted and non-stunted individuals. The data acquired from this study can be used to formulate policies to prevent stunting through targeted interventions for adolescents. Finally, a better understanding of adolescent health could lead to improved strategies to decrease the number of stunted individuals in the next Indonesian generation.

## 1 Introduction

Stunting is defined as impaired growth and development due to chronic malnutrition. Based on UNICEF/World Health Organization (WHO)/World Bank Joint Child Malnutrition data, the global prevalence of stunting among children younger than 5 years was 22.3% in 2022, and 52% of these stunted children were Asian. The same study showed that Indonesia faces three burdens due to malnutrition: stunting; wasting; and obesity (1). According to Indonesia's National Basic Health Research Survey in 2018, the prevalence of stunted children aged 5–12 years was 23.6% (2). Although the prevalence had declined (from 30.7% in 2013) (3), the prevalence of stunted children in Indonesia was still considered among the highest (4). In 2018, this total percentage was higher in rural areas (28.8%) than in urban areas (19%) (2). The NCD-RisC study, which entailed a model-based estimate study of 200 nations, estimated that Indonesian children were 5–10 cm shorter than the WHO standard (5).

In addition to the possibility that children will never reach their full height potential, stunting has long-term effects, such as impaired intellectual ability and poor educational performance, resulting in these adults being less productive and earning lower wages than non-stunted individuals (6). Furthermore, early childhood stunting increases the likelihood of developing obesity (7, 8). The NCD-RisC study indicated that between the ages of 5 and 19 years, the body mass index (BMI) of Indonesian children, particularly girls, increases (5). This condition has become more challenging since obesity is an established risk factor of non-communicable diseases (NCDs) (9). Moreover, during the COVID-19 pandemic, difficulties in obtaining access to nutrient-rich foods and healthcare facilities, physical inactivity, and socioeconomic effects may have increased the prevalence of stunting and overweight/obesity among children (4). In Indonesia, the initiatives aimed at preventing stunting primarily consist of dietary interventions tailored for children aged 5 years and younger (10). However, this stage is regarded as delayed, since stunting is influenced by intrauterine growth restriction (IUGR) that occurs during pregnancy (11). Moreover, stunting is mostly irreversible once the first 1,000 days of life have passed, leading to an intergenerational cycle of stunted growth and development (12).

A study conducted in Indonesia revealed that early life stunting was significantly inversely associated with obesity in a 7-year cohort, but the same data did not reveal any significant associations in a 14-year cohort, with a puberty effect as a potential explanation (13). The pubertal phase is one of the two fastest phases of linear growth beside the first 2 years of life. The time of peak linear growth of adolescents in Western countries is 11.5 years in girls and 13.5 years in boys (14). Pubertal timing is important in determining the final adult height, as the earlier onset of pubertal growth is usually associated with shorter adults. The extent to which the pubertal growth spurt offers a chance for stunted children to “catch-up” and grow to their potential height and the required nutritional contexts in older childhood or early adolescence that promote healthy growth during puberty are unknown (14, 15).

In Indonesia, there have been no research studies that have carefully measured growth, development, and nutritional status across the pubertal years of older childhood and early adolescence. Thus, Indonesia is an important country in which to explore these research questions because of its high rates of stunting in earlier childhood and its growing burden of overweight individuals and obesity. The implications for health and nutrition policy are significant. Therefore, the aim of the present study is to investigate comprehensively the factors associated with stunting in rural and urban children as they pass through adolescence.

This paper outlines the development of a protocol for the first cohort study in Indonesia to describe the trajectory of growth, development (cognitive), pubertal development, psychosocial, mental health, health behavior, as well as the nutritional assessment in a representative population cohort of stunted and non-stunted children in urban and rural areas across three provinces in Indonesia. These individuals will then undergo a series of examinations in which key factors, such as nutritional intake, growth, psychosocial factors, mental health, pubertal development, and endocrine profile, will be assessed annually. The growth profile comprises individuals’ weight, height, and BMI. The nutritional assessment comprises individuals’ waist circumference, middle-upper arm circumference (MUAC), hand-grip strength, body fat percentage, and food intake. The psychosocial and mental health assessment will entail evaluations of behavioral issues, symptoms of depression, quality of life, sleep patterns, anxiety disorders, and parenting style through specific questionnaires. Finally, the pubertal stage will be evaluated using a self-report questionnaire on the puberty stage. In this research, there are also some cross-sectional data, including cognitive performance, hair zinc levels, vitamin D levels, bone mineral density (BMD), and bone age. This research is a collaborative study among the University of Indonesia (UI), Gadjah Mada University (UGM), and Airlangga University (UNAIR), and it involves multiple disciplines. Individuals at The University of Melbourne (UniMelb), a partner university, will assist in monitoring the research. The outcome of this study will provide crucial data for government officials and academics in formulating health policies aimed at preventing stunting and non-communicable diseases in adolescents.

### 1.1 Research objectives

The primary objective of this study is to describe the trajectory of growth, development, behavior, and pubertal timing in a representative population cohort of children in urban and rural areas in Indonesia, to better understand the effect of stunting and to assess the influence of the current nutritional status on pubertal development to guide future health policies. Moreover, the secondary objectives were as follows:

• to investigate the growth profile of stunted versus non-stunted children as they progress through puberty;
• to investigate the trajectory of development (cognitive, psychosocial, risk-taking behavior, and mental health) of stunted versus non-stunted children as they progress through puberty;
• to investigate the timing of puberty in stunted versus non-stunted children, and to examine how this varies by current nutritional status (underweight, normal weight, overweight, obese);
• to investigate the impact of nutritional status and other related factors in children in terms of non-communicable disease and stunting;
• to investigate the impact of nutritional assessment and other related factors in children in terms of growth, development, and timing of puberty;
• to explore the impact of the COVID-19 pandemic on individuals’ growth and nutritional status, developmental trajectories (cognitive, psychosocial, mental health), health behavior (sleep pattern, risk-taking behavior), and puberty stage in stunted versus non-stunted children through adolescence.

## 2 Methods and analysis

### 2.1 Study design

The primary design of this study is a multidisciplinary prospective cohort. The growth profile, developmental trajectories, puberty status, and nutritional assessment will be tracked in this cohort. Some cross-sectional studies of several variables, i.e., cognitive function using an intellectual quotient (IQ) test, Internet addiction, hair zinc levels, vitamin D levels, bone age, and bone mineral density, will also be conducted during this cohort study.

### 2.2 Study setting

This research is a multidisciplinary study that includes UI as the leading university, together with UGM, UNAIR, and UniMelb, the latter of which is a partner university. We started this study after we received ethical approval through 2025. This school-based study will collect data from individuals in elementary schools in three provinces, i.e., DKI Jakarta, DI Yogyakarta, and East Java. The University of Indonesia will be responsible for collecting data from DKI Jakarta to represent the urban population, Gadjah Mada University will collect data from DI Yogyakarta, and Airlangga University will collect data from East Java to represent the rural population. Rural areas were defined according to the percentage of the population living in poverty in a district or city as determined by Central Agency on Statistics of Indonesia in 2020–2021.

### 2.3 Participant criteria

The participants will be Indonesian children, 8-year-old girls and 9-year-old boys, and will be assigned to either the stunted or non-stunted group. Individuals are considered stunted if their height-for-age is less than −1.64 standard deviations (<−1.64 SD) or below the 5th percentile from the growth standard median using the 2007 WHO Reference for individuals aged 5–19 years. In this research, stunted individuals are considered to be the exposure variable. The exclusion criteria for this study are children who have a syndrome or chronic illness that can affect their growth, development, or pubertal status. Participants who do not engage in one or more examination processes for any reason, despite their parents having signed an informed consent form, refuse to continue the study, or cannot be contacted, will be considered as having dropped out.

### 2.4 Recruitment and data collection

The parents of the students will be given 1 week to consider their children's involvement as participants in this study. Once they agree, parents will be asked to sign the informed consent form. Approximately 1,200 students will be screened to recruit 280 stunted participants and 280 non-stunted participants for this research, as the prevalence of stunted children in Indonesia in 2018 was in the range of 25%–30%. The screening will be performed in at least 20 elementary schools in DKI Jakarta, 10 elementary schools in DI Yogyakarta, and 10 elementary schools in East Java. These schools will be chosen using clustered random sampling.

In the first year of this study, before the formal experiments, online and offline training will be provided for all investigators and enumerators for all procedures of the study and the RedCap application. The anthropometric measurements of all 8-year-old girls and 9-year-old boys will be performed by trained enumerators in the school health clinic. Each participant will provide basic demographic information, such as his or her name, date of birth, and sex. All the participants’ anthropometric data will be recorded in the RedCap application by the enumerators and categorized as stunted or non-stunted by research assistants and investigators. The recruitment of 280 stunted participants will be conducted consecutively due to the geographical challenges in reaching rural areas; it is more difficult to recruit stunted individuals from urban areas since the percentage of stunted children in urban areas is lower than in rural areas. Moreover, 280 non-stunted children will be recruited using simple random sampling with sex matching from the first 20 schools. If the required sample size of stunted participants has not been reached after recruiting individuals from the first 20 schools, another randomly selected school on the list will be included in the screening process. Individuals will then undergo a nutritional status assessment in which their waist circumference, MUAC, body fat percentage, and hand-grip strength are measured. Then, enumerators will conduct interviews with the participants and their parents regarding several questionnaires for developmental, psychosocial, mental health, and pubertal aspect assessments. Nutritional assessments and questionnaire interviews will be conducted by different enumerators who are unaware of the children's height. To prevent potential recall bias and missing data, the enumerator will visit the participant's house on two consecutive occasions, given the abundance of questionnaires. All required anthropometric exams, nutritional assessments, and questionnaires will be repeated annually.

**Figure 1:**
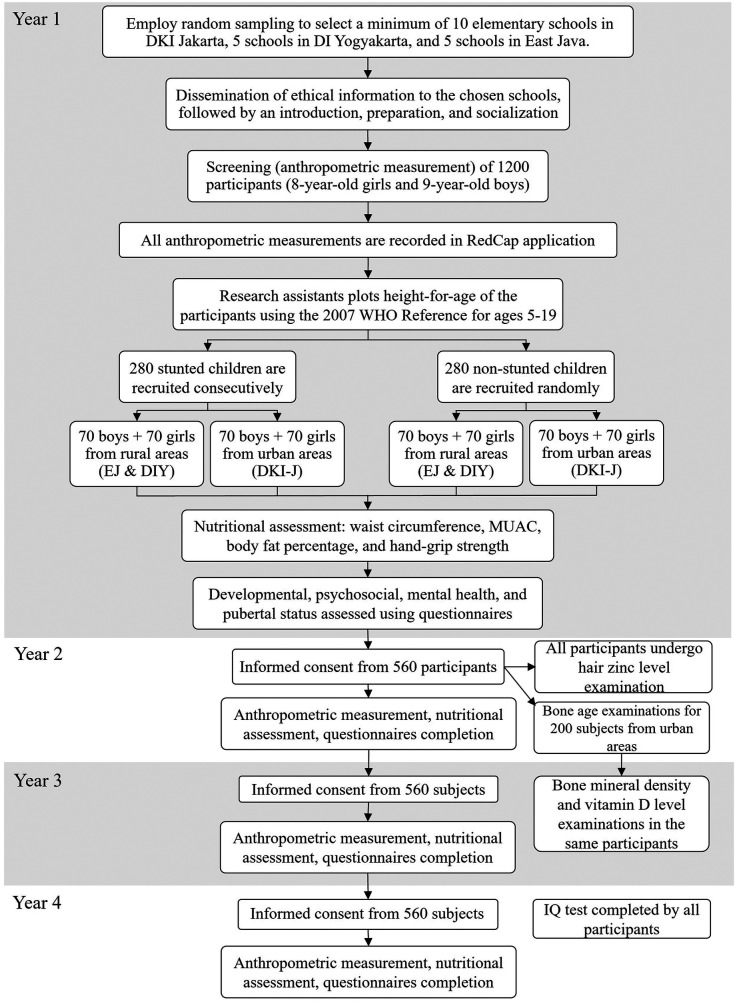
Flow diagram of the study participants.

In the second year of the study, in addition to the anthropometric assessments, nutritional assessments, and questionnaires, all participants will undergo examinations to determine the hair zinc level. Furthermore, participants’ parents from the urban area (DKI Jakarta) will be given an informed consent form for bone age examinations. If the parents agree, a minimum of 100 participants from each stunted and non-stunted group will be chosen for this assessment using simple random sampling. In the third year, these same participants will then undergo vitamin D level and bone mineral density examinations, which will be performed in Cipto Mangunkusumo Hospital, Jakarta. After that, the participants will be followed up and asked to visit the hospital on the determined date. In the third and fourth years, all participants will be asked to complete the Kuesioner Diagnosis Adiksi Internet (KDAI) questionnaire to assess Internet addiction. Finally, in the fourth year of the study, all individuals will complete an IQ test. The IQ test will be administered by a psychologist from each university. A flow diagram of this study can be seen in Figure 1.

### 2.5 Procedure and outcome measure

This study's outcome metrics cover a wide range of factors. Table 1 shows the variables that will be assessed.

**Table 1:** Variables and study years.

Variables	Year 1	Year 2	Year 3	Year 4
Growth profile: • Weight • Height • Height-for-age • Weight-for-height • BMI	✓	✓	✓	✓
Nutritional assessment: • Waist circumference • Middle-upper arm circumference • Hand-grip strength • Body fat percentage determined using the Bioimpedance Analysis method • Food intake using the FFQ	✓	✓	✓	✓
Self-rated puberty stage according to the PDS	✓	✓	✓	✓
Psychosocial and mental health: • Behavioral problems indicated in the SDQ • Depressive symptoms indicated in the CDI • Quality of life indicated in the PedsQL • Sleep quality indicated in the PSQI • Anxiety disorder indicated in the GAD-7 • Parenting style indicated in the PSDQ	✓	✓	✓	✓
Lifestyle changes and family history during the COVID-19 pandemic	✓	✓		
COVID-19 immunization status	✓			
Bone age examination		✓(DKI-J)		
Hair zinc level		✓		
Vitamin D level			✓(DKI-J)	
Bone mineral density			✓(DKI-J)	
Internet addiction indicated in the KDAI questionnaire			✓	✓
Cognitive function				✓

DKI-J, only for participants from DKI Jakarta.

#### 2.5.1 Anthropometric measurement

Anthropometric data consist of weight and height measurements, followed by BMI calculation. For weight measurements, the participant will be asked to stand still on the weighing scale, with his or her feet slightly apart or on the marks provided by the instrument, after removing their shoes and stockings and while wearing the minimum school uniform (16). The study will use a Tanita RD 953 weighing scale. The participant's weight will be recorded to the nearest 0.01 kg unit. For height measurements, the participant will be instructed to stand straight with their heals, calves, buttocks, scapula, and the back of the head touching the vertical side of the stadiometer (16). This study will use a SECA 213 stadiometer. The participant's height will be recorded to the nearest 0.1 cm. Next, the research assistants will calculate the BMI of the participant by dividing the child's weight (in kg) by the square of the person's height (in m^2^). Both the height and BMI will be plotted to create height-for-age and BMI-for-age curves, respectively, using the 2007 WHO Reference (17). Stunting is determined when the height-for-age measurement is less than −1.64 standard deviations below (<−1.64 SD) or below the 5th percentile from the growth standard median, based on the 2007 WHO Reference for people aged 5–19 years.

#### 2.5.2 Waist circumference

Participants will be told to stand upright with their feet together and gently cross their arms over their chests. The enumerator will stand on the participant's right side, palpate the right ileum, and draw a horizontal line on the uppermost border of the iliac cress. After that, the midpoint of the inferior margin of the final rib and the horizontal line of the uppermost iliac crest that was previously drawn will be determined. A constant tension tape will be wrapped around the waist at the marked midpoint. The other enumerator will ensure that the tape on the left is straight and parallel to the floor (18). The constant tension tape used in this research is SECA 201 constant tension tape. After normal exhalation, the participant's waist circumference will be recorded to the nearest 0.1 cm.

#### 2.5.3 Middle-upper arm circumference

The arm circumference will be measured on the right arm at the level of the upper-arm midpoint. First, the upper arm length will be measured from the uppermost edge of the posterior border from the acromion process to the tip of the olecranon, with the right arm bent 90° at the elbow and the right palm facing up. The midpoint will be marked with a marker. The participant will then be asked to stand upright facing the enumerator with the right arm hanging loosely. The measuring tape will be wrapped perpendicular to the long axis of the upper arm and around the arm at the midpoint. The measuring tape will be pulled until the zero end sits below the measurement value (19). The arm circumference will be measured to the nearest 0.1 cm.

#### 2.5.4 Hand-grip strength

The hand-grip strength of each participant's dominant hand in daily life will be measured. Participants will be instructed to squeeze a Jamar hand dynamometer with their maximum strength after adjusting the handle to the proper position and rotating the red peak-hold needle counterclockwise to zero (20). The highest peak-hold value will be recorded to the nearest 0.1 kg.

#### 2.5.5 Body fat percentage

Body fat percentage will be measured concurrently with the participant's weight using a Tanita RD 853 scale. The body fat percentage will be displayed via phone application, and the enumerator will record the fat percentage to the nearest 0.1%.

#### 2.5.6 Nutritional intake

The nutritional intake of processed foods will be evaluated using a modified food frequency questionnaire. The questionnaire was created by a Pediatric Nutrition and Metabolic Disease specialist in Bahasa Indonesia and is validated for children aged 8–12 years.

#### 2.5.7 Stage of puberty

The stage of puberty will be determined using the Pubertal Developmental Scale (PDS) (21). Participants will be asked to complete the questionnaire after the anthropometric and nutritional assessments in school. If a participant is unable to read, enumerators will help by reading the questionnaires and answers to the individual.

#### 2.5.8 Behavioral problems

Behavioral problems will be measured using the Strength and Difficulty Questionnaire (SDQ) for people aged 4–17 years; this questionnaire has been validated in Bahasa Indonesia. The questionnaire consists of five domains, each with five items. The domains are emotional (E), conduct problems (C), hyperactivity (H), peer problems (P), and prosocial behavior (Pr). Enumerators will interview the participant's parents and have them answer 25 questions. For each item, the response options are not true, somewhat true, or certainly true, and will be scored as 0, 1, or 2, respectively. The score of each item is summed according to its domain. If the sum of Pr, E, C, H, and P domains are 5, 4, 3, 6, and 3, respectively, the participant is classified as borderline. If the cumulative score for each domain is greater than the borderline threshold, the participant is considered abnormal; if the cumulative score is less than the borderline threshold, the participant is considered normal (22, 23).

#### 2.5.9 Depressive symptoms

Potential depression symptoms are detected using the Children's Depression Inventory (CDI). This questionnaire has been validated by the Pediatric Department of Cipto Mangunkusumo Hospital and will be completed by all participants after completing the PDS questionnaire during the classroom data collection session. The participants will be instructed to complete the CDI in less than 15 min by placing an “X” next to the sentence that best describes their condition over the previous 2 weeks. The sum of all items’ scores will be compared to the cutoff. If the participant's total score exceeds the cutoff point (13), potential depression is indicated for the participant (24, 25).

#### 2.5.10 Quality of life

Quality of life will be measured using the version of the Pediatric Quality of Life Inventory (PedsQL) Core 4.0 questionnaire for children aged 8–12 years. The PedsQL 4.0 Generic Core Scale has been validated for use in Bahasa Indonesia by Sitaresmi et al. For this questionnaire, all individuals will answer 5-point Likert scale questions, and the sum for every subscale and the total scale will be calculated (26).

#### 2.5.11 Sleep quality

Sleep quality will be measured using the Pittsburgh Sleep Quality Index (PSQI) questionnaire. The version validated in Bahasa consists of 19 self-reported questions and 5 bedpartner-reported questions. The self-rated scale of 19 questions has response values in the range of 0–3. Moreover, the five questions scored by the bedpartner will be excluded from the analysis; they will be utilized solely for medical information. When the total score is ≥21, difficulties in all areas are indicated (27).

#### 2.5.12 Anxiety disorders

Any potential generalized anxiety disorder (GAD) symptoms will be detected using the Generalized Anxiety Disorder-7 (GAD-7) questionnaire. The overall response to the 3-point Likert questions will be grouped into four categories, with total scores of 0–4 representing low anxiety, 5–9 representing mild anxiety, 10–14 representing moderate anxiety, and 15–21 representing severe anxiety (28). The questionnaire of GAD-7 for pediatric participants will be validated in Bahasa Indonesia before examination.

#### 2.5.13 Parenting style

Parenting style will be assessed using the Parenting Style and Dimension Questionnaire (PSDQ). The PSDQ was validated in Bahasa Indonesia in 2018 and has undergone reliability test in more than 2,000 individuals (29). This study will administer the 31-item 5-point Likert scale PSDQ to all parents whose children are included in this study.

#### 2.5.14 Lifestyle changes and family history during the COVID-19 pandemic

The questionnaire about lifestyle changes and family history during the COVID-19 pandemic analyzed the health statuses of individuals and their families throughout the preceding 2 years of the pandemic. This questionnaire was derived from the Mental Health Survey conducted in Indonesia by Wiguna et al. (30). Although this will be a univariate study, the questionnaire will be validated, and reliability will be tested on 30 pediatric individuals before the examination session. The questionnaire will include whether the child or anyone in his or her family history was positive for COVID-19, lifestyle changes during the COVID-19 pandemic, support from family and friends, and access to information about mental healthcare.

#### 2.5.15 COVID-19 vaccination status

This questionnaire will be administered only to individuals' parents in the first year of the study. The questionnaire asks questions about the child's history of COVID-19 vaccination, the vaccine name, the number of doses administered, and the parents’ attitude toward the COVID-19 vaccine. The vaccination status of the child will be verified using the Peduli-Lindungi application, which is available nationwide for those who have received a COVID-19 vaccine.

#### 2.5.16 Hair zinc level

Hair zinc level will be examined in the second year of the study. The enumerator will remove a single hair strand from the occipital-nuchal region of the head and then the first 3 cm from the hair root will be stored in chemical-free containers (31, 32). The zinc content of the hair will be determined in Jakarta District Laboratories using the inductively coupled plasma–optical emission spectrometry (ICP–OES) method and reported to the nearest 0.1 μg/g.

#### 2.5.17 Bone age examination

Bone age examination will be performed by taking x-ray images of participants’ left wrists and hands in the anteroposterior (AP) position in the second year of the study. This bone age examination will be performed by a pediatrician specializing in radiology imaging at Dr. Cipto Mangunkusumo Hospital. The images will be reviewed by pediatric endocrinologists in a blinded manner. Any disagreement between two pediatric endocrinologists will be resolved by including the interpretation of a third pediatric endocrinologist. Bone age will be recorded in months.

#### 2.5.18 Vitamin D level

Vitamin D levels of participants from the urban area will be examined in the third year of the study. A minimum of 3 mL of blood will be drawn from the cubital vein to the SST by a phlebotomist. The 300 μL serum will be centrifuged and examined using a Liaison instrument with chemiluminescent immunoassay (CLIA) method (33–35). This laboratory examination will be conducted at the Prodia Laboratories branch in DKI Jakarta, which is certified by ISO 9001 2008 and ISO 15189.

#### 2.5.19 Bone mineral density

The BMD of 200 randomly selected participants from urban areas will be measured in the third year of the study. Before the examination begins, the participant will be asked to remove any accessories and only wear an examination gown. The bone mineral density of the lumbar vertebrae, femur, and antebrachial bone (radius and ulna) will be measured by using dual energy x-ray absorptiometry (DXA) in a 10–20-min session. This examination is non-invasive since the radiation exposure dose is low. This examination will be performed in the Radiology Department of Dr. Cipto Mangunkusumo Hospital. The result will be recorded to the nearest 0.1 T-score.

#### 2.5.20 Internet addiction

The KDAI is a questionnaire to identify Internet addiction. It was developed in Indonesia as a screening tool for Internet addiction within the past 12 months. The KDAI is answered using a Likert-type scale from 1 to 6 with a score of 1 indicating very rare, 2 signifying rare, 3 indicating seldom, 4 suggesting often, 5 indicating very often, and 6 signifying always (36). This questionnaire will be administered in the third and fourth years of the study and will be completed by the children themselves if they are aged 10 years. If the total score is ≥108, the participant is considered to be addicted to the Internet.

#### 2.5.21 Cognitive function

Cognitive function will be assessed using an IQ test. This test will be performed before the physical examination in the fourth year of the study. The IQ test will be developed by a team of certified psychologists from the Psychology Clinic Universitas Indonesia in collaboration with psychologists from UGM and UNAIR. The results will be classified according to the Wechsler Intelligence Scale for Children—Fifth Edition (WISC-V), i.e., the IQ ranges of <70, 70–79, 80–89, 90–109, 110–119, 120–129, and ≥130 will be classified as extremely low, very low, low average, average, high average, very high, and extremely high, respectively.

### 2.6 Sample size

This school-based study will recruit a total of 560 individuals. Since there has been no research on multiple parameters of stunted children, the sample size was derived using a rule of thumb with an estimated dropout rate of 30% to ensure that enough data are collected for variables, i.e., weight, height, height-for-age, BMI, waist circumference, MUAC, hand-grip strength, body fat percentage, food intake, pubertal status, behavioral problems, depressive symptoms, quality of life, sleep quality, anxiety disorder, parenting style, hair zinc level, cognitive function, lifestyle changes during the COVID-19 pandemic, and Internet addiction. Due to limited available examinations, only approximately 100 participants from each stunted and non-stunted group in DKI Jakarta will undergo bone age, vitamin D, and bone mineral density examinations; these examinations will be performed on the same individuals. This subsample size was determined by comparing categorical variables from previous studies in Asia (35, 37, 38).

### 2.7 Data storage and statistical analysis

All physical examinations, questionnaires, and laboratory results will be coded using the individual’s identity (ID) assigned to the RedCap application from UI. An enumerator who is not participating in the anthropometric data collection process will record these results for each participant, and another enumerator will double-check the encoded data. In addition, only the principal investigator has access to participant identifiers in the RedCap application. The data will be analyzed using the Statistical Package for the Social Sciences (SPSS) version 24.0 for Windows by a biostatistician who is not involved in data collection and does not have access to participant identifiers.

Univariate analysis data will be presented as numbers and percentages for categorical data. Numerical data will be presented as means ± SDs if the data are normally distributed, and as medians (min–max) if they are not normally distributed. Trends in the evaluation data collected yearly, such as growth, psychosocial, nutrition, and pubertal aspects, will be presented as graphs and will be analyzed as repeated measurements. The mean difference (of BMI, waist circumference, MUAC, hand-grip strength, body fat percentage, bone age, hair zinc level, vitamin D level, and IQ) between stunted and non-stunted individuals will be analyzed using the independent T-test if the data are normally distributed (*P* < 0.05). Otherwise, the Mann–Whitney U test will be performed when the data are not normally distributed. Moreover, we will use the chi-square test or Fisher’s exact test (if the data do not meet the chi-square criteria) to analyze the associations among BMI-for-age, stage of puberty, behavioral problems, depressive symptoms, sleep quality, GAD symptoms, parenting style, and bone mineral density in stunted participants. *P*-values <0.05 indicate statistical significance. We determined 80% as the power of this research and used this as our sample size calculation since it is acceptable in medical research and the sample size we obtained through calculation is still within our research budget. If we used a power of 90% and in the sample size calculation, the number will be larger and exceed our grant research budget. In addition, a multivariate analysis will be conducted using a logistic regression test to determine the dominant variables associated with the stunted condition adjusted for confounding factors.

## 3 Discussion

According to a health survey by the Ministry of Health for the Republic of Indonesia, the prevalence of stunting in Indonesia in 2022 decreased to 21.6% (39). Despite a downward trend, estimates by UNICEF/WHO/World Band models still regarded the prevalence of stunting in Indonesia as very high (1). Therefore, the president of Indonesia has established goals to reduce the prevalence of stunting to 14% by 2024 (40). These goals will be achieved through the implantation of five primary strategies. The initial approach entails work at the government at the provincial, regional, and district levels, evidenced by the established of new regulations. The second strategy involves providing better education on lifestyle modifications through a nationwide campaign and making this information available across all forms of media. The third strategy involves enhancing targeted intervention convergence and responsive intervention, including data-driven planning and budgeting, as well as program convergence, including the management of programs conducted throughout the first 1,000 days of life. The fourth plan aims to provide access to nutritious food and promote food security. The last approach involves improving the monitoring and evaluation processes to create a foundation that ensures the provision of high-quality services, promotes accountability, and facilitates learning (10). The initiatives consist primarily of targeted nutrition interventions for children aged younger than 5 years (40). However, this is considered a late period given that IUGR during pregnancy contributes to stunting (11). Preconception, the prenatal period, and infant-toddlerhood are all critical periods during which the mother's nutrition and health status play a significant role in preventing stunting (41). Thus, it is imperative to establish a prevention program well before the onset of stunting by targeting adolescents.

Though previously ignored due to perceptions of adolescence as a healthy phase of life, the extent to which health trajectories and health behaviors can change during adolescence is becoming increasingly appreciated. Adolescence is a period during which individuals undergo dramatic changes, such as eating unhealthy diets, being physically inactive, and becoming addicted to the Internet, among other risky behaviors (42, 43). Several eating behaviors were also identified in a systematic review of adolescent girls’ eating behaviors in countries with a high prevalence of stunting in children aged younger than 5 years. These behaviors included eating a low diversity diet, engaging in an unhealthy dietary pattern, craving-induced eating, restrained eating, losing weight, and showing symptoms of eating disorders. Inadequate dietary intake and micronutrient deficiencies, which are indicative of a low-diversity diet, can lead to adverse birth outcomes, such as contracted pelvis, delayed puberty, and stunted growth in the newborn (44).

Moreover, adolescence is a crucial period as it is marked by the occurrence of puberty. A study conducted in Indonesia revealed that early-life stunting was significantly inversely associated with being overweight/obese in a 7-year cohort, but the same data did not reveal any significant associations in a 14-year cohort, and the effect of puberty was a potential explanation (13). Pubertal timing is an important factor in one's final adult height as earlier onset of the pubertal growth spurt is usually associated with shorter adults (45). The extent to which the pubertal growth spurt offers the chance for stunted children to “catch-up” to their potential height and the required nutritional contexts in older childhood or early adolescence that promote healthy growth during puberty are unknown (14, 15). These factors are important to know as puberty is also associated with risks. In Latin America, overly enthusiastic feeding programs in stunted young children have resulted in early obesity, with obvious implications for health and nutrition policies (46). Puberty is a multifaceted developmental process that begins in late childhood with a cascade of endocrine changes that ultimately lead to sexual maturation and reproductive capability. The transition through puberty is marked by an increased risk for the onset of a variety of health problems, particularly those related to the control of behavior and emotion. Early-onset puberty is associated with a greater risk of cancers of the reproductive tract and cardiovascular disease (47). Hence, this research will provide longitudinal data on the growth, trajectory development, nutritional status, and other relevant factors related to NCDs and the pubertal aspect of stunted children in comparison to non-stunted children as they progress through adolescence. In addition, the tests will be performed in various laboratories to measure hair zinc levels and vitamin D levels, as well as radiographic examinations to assess bone age and bone mineral density. These tests results provide further data related to stunting in Indonesia.

Rather than old views of adolescence as the end of childhood, newer views appreciate adolescence as the start of the next generation. The Lancet Commission on Adolescent Health and Wellbeing noted the importance of investing in interventions in adolescence to achieve a “triple dividend”—of better health during adolescence itself, of better adult health as healthy adolescents mature, and of better health for the next generation when today's adolescents choose to parent (48). Adolescent girls, in particular, will eventually bear children, raise a family, and nurture a new generation of Indonesians; their healthy, educated presence is vital for a family's overall health. Therefore, the findings of this study will serve as a resource for the government to use to formulate health policies aimed at preventing stunting far before the start of the 1,000-day period of life. The proposed interventions include the following: promoting healthy eating behaviors and dietary intake to both parents and adolescents; implementing a specialized module in schools that focuses on dietary intake and healthy eating behavior; conducting screenings and providing early interventions for children at high risk of NCDs; implementing a stricter system to prevent child marriage during adolescence; disseminating information about the risks of stunting during pre-wedding preparations via public health centers; referring individuals with abnormal psychosocial and mental health questionnaire responses, vitamin D levels, or hair zinc levels to the nearest public health center; and proposing a specific system for managing the results of zinc and vitamin D level assessments. Through this research, our aim is to enhance the understanding of stunting and NCDs by specifically focusing on adolescents, a previously unexplored demographic. By doing so, we intend to disrupt the intergenerational cycle of malnutrition.

A limitation of our study is that the research will only be conducted in Java Island. However, approximately 56% of Indonesia's population resides in Java. Furthermore, some of the variables will have cross-sectional data due to a limited budget. Another limitation of this study is that the participants are only to be observed until the age of 12 years or throughout the early stages of adolescence as determined by the grant timetable. However, given that this is a novel cohort study, the evaluation of this cohort could be extended by another grant or research project, allowing more data on adolescents to be collected. Moreover, potential sources of bias include recall bias. However, to prevent this, the questionnaires will be completed over multiple sessions, allowing parents sufficient time to complete them. The confounding variables comprise the genetic background and socioeconomic status of the families. The potential influence of genetics as a confounding variable will be addressed by administering the questionnaire at the participants’ residences to collect data on their potential influence of genetics on their height. The potential factors contributing to discrepancies may include cultural variations, resource disparities, and distinct differences in eating patterns in Jakarta and other regions in Java.

## Ethics statement

The studies involving humans were approved by Faculty of Medicine, University of Indonesia. The studies were conducted in accordance with the local legislation and institutional requirements. Written informed consent for participation in this study was provided by the participants’ legal guardians/next of kin.
